# Robust dose planning objectives for mesorectal radiotherapy of early stage rectal cancer – A multicentre dose planning study

**DOI:** 10.1016/j.tipsro.2019.09.001

**Published:** 2019-10-15

**Authors:** Ane L. Appelt, Ellen M. Kerkhof, Lars Nyvang, Ernst C. Harderwijk, Natalie L. Abbott, Mark Teo, Femke P. Peters, Camilla J.S. Kronborg, Karen-Lise G. Spindler, David Sebag-Montefiore, Corrie A.M. Marijnen, On behalf of the STAR-TREC collaborative group

**Affiliations:** a Leeds Institute of Medical Research at St James’s, University of Leeds and Leeds Cancer Centre, St James’s University Hospital, Leeds, UK; b Department of Radiotherapy, Leiden University Medical Center, Leiden, the Netherlands; c Department of Oncology, Aarhus University Hospital, Aarhus, Denmark; d Radiotherapy Trials Quality Assurance Group, Velindre Cancer Centre, Cardiff, UK; e Leeds Cancer Centre, St James’s University Hospital, Leeds, UK

**Keywords:** Rectal neoplasms, Radiotherapy, Intensity-modulated, Organ preservation

## Abstract

**Background and purpose:**

Organ preservation strategies are increasingly being explored for early rectal cancer. This requires revision of target volumes according to disease stage, as well as new guidelines for treatment planning. We conducted an international, multicentre dose planning study to develop robust planning objectives for modern radiotherapy of a novel mesorectal-only target volume, as implemented in the STAR-TReC trial (NCT02945566).

**Materials and methods:**

The published literature was used to establish relevant dose levels for organ at risk (OAR) plan optimisation. Ten representative patients with early rectal cancer were identified. Treatment scans had mesorectal target volumes as well as bowel cavity, bladder and femoral heads outlined, and were circulated amongst the three participating institutions. Each institution produced plans for short course (SCRT, 5 × 5 Gy) and long course (LCRT, 25 × 2 Gy) treatment, using volumetric modulated arc therapy on different dose planning systems. Optimisation objectives for OARs were established by determining dose metric objectives achievable for ≥90% of plans.

**Results:**

Sixty plans, all fulfilling target coverage criteria, were produced. The planning results and literature review suggested optimisation objectives for SCRT: *V*_10Gy_ < 180 cm^3^, *V*_18Gy_ < 110 cm^3^, *V*_23Gy_ < 85 cm^3^ for bowel cavity; *V*_21Gy_ < 15% and *V*_25Gy_ < 5% for bladder; and *V*_12.5Gy_ < 11% for femoral heads. Corresponding objectives for LCRT: *V*_20Gy_ < 180 cm^3^, *V*_30Gy_ < 130 cm^3^, *V*_45Gy_ < 90 cm^3^ for bowel cavity; *V*_35Gy_ < 22% and *V*_50Gy_ < 7% for bladder; and *V*_25Gy_ < 15% for femoral heads. Constraints were validated across all three institutions.

**Conclusion:**

We utilized a multicentre planning study approach to develop robust planning objectives for mesorectal radiotherapy for early rectal cancer.

## Highlights

• Mesorectal-only RT for early rectal cancer requires dedicated planning guidelines.
• A multi-centre study was conducted to identify planning objectives.
• Robust optimisation objectives for organs at risk are presented.

## Introduction

Radiotherapy for rectal cancer has traditionally been used in the neoadjuvant setting, prior to radical surgery. Standard total mesorectal excision (TME) surgery can, however, result in significant morbidity and mortality. There is consequently an increasing interest in organ preservation and non-surgical management strategies. This is currently primarily considered when the standard of care involves pre-operative chemoradiotherapy, generally in patients with a moderate or high risk of local recurrence, who have a complete clinical response to treatment.

In early stage rectal cancer with a low local recurrence risk after radical surgery, patients do not usually receive preoperative radiotherapy. The benefit of organ preservation approaches compared with radical surgery are being evaluated in this setting in the international phase III STAR-TREC trial (ClinicalTrials.gov Identifier: NCT02945566) [1]. This trial focuses on early stage cancer and requires a re-evaluation of target volumes and treatment planning principles. The rationale and development of a novel mesorectum-only target volume for early rectal cancer has been discussed elsewhere [1], [2], but dose planning techniques for this approach have not yet been described.

In STAR-TREC, we aim to spare the pelvic normal tissues from unnecessary irradiation and minimise acute toxicity [3] and late toxicity [4]. This is achieved with the use of intensity modulated radiotherapy or arc therapy and pre-specified treatment planning objectives for the relevant organs at risk. As for any other introduction of radiotherapy regimens and volumes in novel settings, identification of treatment planning objectives can prove challenging, as limited data are available. Alternative approaches may be needed, in the absence of clinical outcome data to drive OAR constraints, to reduce dose to normal tissue as far as reasonably achievable.

The purpose of the current study was to establish organs at risk (OAR) dose metric optimisation objectives for evaluation of inversely planned IMRT mesorectum-only treatment plans. These would aim to ensure that a majority of treatment plans are sufficiently conformal with respect to the OARs. We set out to develop and subsequently confirm the robustness of the optimisation objectives across multiple dose planning systems and individual planners.

## Materials and methods

### Literature search

A systematic search was conducted on PubMed, using a combination of keywords representing variations of “radiotherapy”, “gastrointestinal toxicity” and “dose-response” or “dose-volume”, published in English language up to May 2018. See Appendix A (supplementary materials, online only) for details. We identified studies reporting correlations between dose metrics and early and late bowel toxicity with external beam radiotherapy, and performed a narrative review of those. A similar search and review were conducted for bladder toxicity (“radiotherapy”, “bladder toxicity” and “dose-response” or “dose-volume”), deliberately focusing on papers describing outcomes after rectal cancer treatment, and for femoral head toxicity. Due to the novel target volume considered here, and variation in normal tissue delineation across the literature, no attempt was made to identify specific dose metric cut-offs in previous publications. We divided the identified dose levels into those relevant for establishment of plan optimisation objectives and those mainly relevant for plan comparisons in the current study.

### Patients

Ten patients with early rectal cancer (T1-3a, N0) were identified from patient records at Leeds Cancer Centre, UK, and were selected to include a range of male and female patient anatomy; patients with high/mid and low tumours; and patients treated in prone and supine position. See Table 1 for patient characteristics. Local Leeds Teaching Hospitals Trust (LTHT) Research & Development approval was obtained for use of patient data.

**Table 1:** Patient characteristics. All continuous measures represent median values with interquartile range in brackets. CTV: Clinical target volume. PTV: Planning target volume. Patients treated in the prone position did not use a belly board.

Patient characteristics	
Gender	4 female/6 male
Treatment position	5 prone/5 supine
Disease stage	1 T1/8 T2/1 T3a 10 N0
T site in rectum	1 high/5 mid/4 low
CTV [cm^3^]	226 (176–238)
PTV [cm^3^]	526 (458–568)
Bowel cavity [cm^3^]	946 (693–1354)
Bowel loops [cm^3^]	381 (144–492)
Bladder [cm^3^]	201 (115–304)
Right femoral head [cm^3^]	169 (129–215)
Left femoral head [cm^3^]	169 (129–216)

Patients were scanned for treatment planning using 5 mm CT slice thickness. Mesorectal-only target volumes (clinical target volume, CTV) were delineated according to the STAR-TREC contouring guidelines (Peters et al., submitted). In brief, this volume includes the mesorectum and pre-sacral lymph nodes at the level of the tumour, two centimetres below and cranially up to the S2-3 interspace level. The lateral lymph nodes and the nodes along the superior rectal artery are excluded. The planning target volumes (PTV) were created using 10 mm craniocaudal, posterior and lateral margins and 15 mm anterior margins. The bowel cavity was outlined using adapted RTOG guidelines, including the abdominal contents but excluding major vasculature, muscles and bones, as well as other pelvic organs (e.g. bladder, prostate, vagina, uterus), extending 2 cm above the superior extent of the PTV and inferiorly to where small bowel or colon is visible. The mesorectum and rectum were excluded from the volume. Due to the uncertainty surrounding optimal bowel definition for pelvic radiotherapy planning, individual bowel loops were additionally contoured as a separate volume, to allow evaluation of dose metrics (but not to be used for plan optimisation). The whole bladder was delineated including urine compartment and bladder wall. The right and left femoral heads were contoured separately, with the caudal extension inferiorly to the lesser trochanter. All outlining was done by an experienced clinical oncologist consultant (who wrote central aspects of the contouring guidelines), with support from a GI radiologist and feedback from other senior trial members. Table 1 contains information regarding target and OAR volumes for the patient cohort.

### Treatment planning and evaluation

Three experienced planners from three major academic radiotherapy centres (St James University Hospital, Leeds, UK; Leiden University Medical Center, Leiden, Netherlands; Aarhus University Hospital, Aarhus, Denmark) produced two plans each for each patient case: (1) Long-course radiotherapy plan of 50 Gy in 25 fractions; (2) Short course radiotherapy plan of 25 Gy in 5 fractions. Each centre used a different planning system: *Eclipse* (Varian Medical Systems), *Pinnacle* (Philips Healthcare), and *Monaco* (Elekta). Planners were asked to produce as conformal plans as possible, while ensuring that they would be deliverable and acceptable in their local clinical practice with respect to physical plan parameters, treatment time, plan robustness, and general clinical setup. Each centre reviewed their treatment plan strategy (beam setup, prioritisation of planning objectives, etc) with a radiation oncologist experienced in rectal cancer treatment. Target planning objectives followed standard ICRU 83 criteria, focusing on full coverage of the target volume with the 95% isodose (*V*_95%_ ≥ 100% for CTV, *V*_95%_ ≥ 99% for PTV), no hotspots (*V*_105%_ ≤ 1% for PTV), and keeping the median target dose (D_50%_) within 2% of the prescription dose. Generally, target volume (PTV) dose homogeneity, coverage and conformity were prioritised over specific OAR sparing.

*Eclipse:* Arc therapy treatment plans consisted of a full dual arc over 358°, delivered using a 15 MV beam. Arcs had control points every 2°. Dose calculation was performed with the Varian Acuros XB algorithm, with 2 mm grid spacing.

*Pinnacle:* Arc therapy treatment plans consisted of a dual partial arc over 268°, to avoid the bowel cavity and the bladder, delivered using a 10 MV beam. Arcs had control points every 4°. Dose calculation was performed with a collapsed cone-based algorithm, with 4 mm grid spacing.

*Monaco:* Arc therapy treatment plans consisted of either a full dual arc or dual partial arcs (45–180° and 315–110°), depending on patient anatomy, delivered using a 6 MV flattening filter free (FFF) beam. Arcs had a maximum of 50 control points per arc and 1 cm minimum segment width. Dose calculation was performed with a Monte Carlo based algorithm, with 3 mm grid spacing and 1% statistical uncertainty.

Dose metrics, as identified in the process described above, were extracted from dose volume histograms (DVH) in each planning system and collated across centres. Descriptive statistics (median and interquartile range, IQR) were summarised for volumes of interest. Dose metric optimisation objectives achievable for a majority of patients were originally identified in a single centre (centre 1) using the following criteria:

(1) For bowel cavity: Objectives chosen to be achievable for at least 80% of plans.
(2) For bladder and femoral heads: Objectives chosen to be achievable for at least 90% of plans.

This expert-based approach ideally identifies objectives which ensure active treatment optimisation for most patients, without failing more than a small minority after plan optimisation; with priority given to bowel relative to bladder and femoral heads.

Subsequently, these criteria were tested across dose planning systems and dose planners, and deemed robust if they fulfilled the above criteria for any individual centres as well as all being achievable for 90% of all plans across centres.

Two conformity indices (CIs) were chosen for plan comparison purposes:

$$CI1=\frac{V_{95\%,PTV}}{V_{95\%,patient}}$$

$$CI2=\frac{V_{50\%,patient}}{V_{\mathrm{total},PTV}}$$

Multiple CIs have been reported in the literature [5]; the ones used here focus on the conformity of high dose to the PTV and the spill-over of median dose levels into surrounding normal tissue.

## Results

### Literature search: OAR dose levels for LCRT

A substantial number of studies reported results for 3D conformal radiotherapy (3D-CRT), the majority of which has been reviewed in [6]. Studies in rectal and anal cancer as well as selected studies in prostate and gynaecological cancer are summarised in Appendix A (supplementary materials, online only).

**Bowel:** For rectal cancer patients, preoperatively treated with 3D-CRT, the absolute bowel volume receiving ≥15 Gy (*V*_15Gy_) has consistently been found to correlate with acute GI toxicity [7], [8], [9], [10], [11]. Studies of rectal [12], anal [13], [14], [15], [16], [17], prostate [18], [19], [20] and gynaecological [21], [22], [23] cancer patients treated with IMRT or arc therapy have found correlations between acute GI toxicity and dose levels from approximately 25–45 Gy, delivered in 25–28 fractions. For late GI toxicity in rectal cancer, the data is very limited. There is some limited evidence that absolute dose volumes in this same range (*V*_30Gy_–*V*_45Gy_) may correlate with late toxicity [6], [24], [25], [26]. One high dose level (*V*_45Gy_) as well as two medium dose levels (*V*_20Gy_ and *V*_30Gy_) were chosen for optimisation objectives. Additionally, *V*_15Gy_ was chosen for plan comparison purposes.

**Bladder:** A single study from the neoadjuvant rectal cancer setting was identified [27]. Appelt et al found that relative volume of the bladder receiving 35 Gy or above (*V*_35Gy_) in 25–30 fractions correlated with acute urinary toxicity. Data from bladder cancer patients indicate that long term functional outcome may also be related to volume of bladder exposed to 45–50 Gy [28], [29]. Consequently, *V*_35Gy_ and *V*_50Gy_ were chosen as optimisation objectives, with *V*_15Gy_ additionally chosen for plan comparison purposes.

**Femoral heads:** Data on dose-volume relationships for femoral heads are extremely sparse [28]. Consequently, a pragmatic decision was made to optimise the volume receiving 50% of the prescription dose (*V*_25Gy_) to prevent lateral dose dumping.

### Literature search: OAR dose levels for SCRT

The vast majority of publications focus on normo-fractioned (1.8–2.0 Gy) treatment (see previous section), with only two papers providing any suggestions for optimisation constraints for SCRT [30], [31]. Due to the lack of reliable data, optimisation constraints were guided by conversions from 1.8 to 2 Gy per fraction to 5 Gy per fraction using the linear quadratic model, with *α*/*β* = 10 Gy for acute toxicity and *α*/*β* = 3 Gy for late toxicity. Details of recalculations can be found in Appendix B (supplementary materials, online only).

**Bowel:** 30 Gy in 25–28 fractions corresponds to 23 Gy (*α*/*β* = 10 Gy) and 18 Gy (*α*/*β* = 3 Gy) in 5 fractions. As 23 Gy additionally provides a suitable optimisation point close to the prescription dose level, *V*_18Gy_ and *V*_23Gy_ were deemed appropriate for optimisation of SCRT plans. To ensure optimisation of medium/low dose levels, *V*_10Gy_ was added to the optimisation objectives, with *V*_12.5Gy_ used for plan comparison purposes only (corresponding to 15 Gy in 25 fractions).

**Bladder:** The same approach as above was used, where 35 Gy delivered in 25–28 fractions corresponds to 25–26 Gy (*α*/*β* = 10 Gy) and 21 Gy (*α*/*β* = 3 Gy) in 5 fractions. Two objectives representing this range (*V*_21Gy_ and *V*_25Gy_) were chosen; with the higher dose level controlling the volume of the bladder receiving prescription dose. *V*_12.5Gy_ was reported for plan comparison.

**Femoral heads:** Volume of femoral heads receiving 50% of the prescription dose (*V*_12.5Gy_) was chosen, as for LCRT.

### Planning results and suggested optimisation objectives

**Fig. 1:**
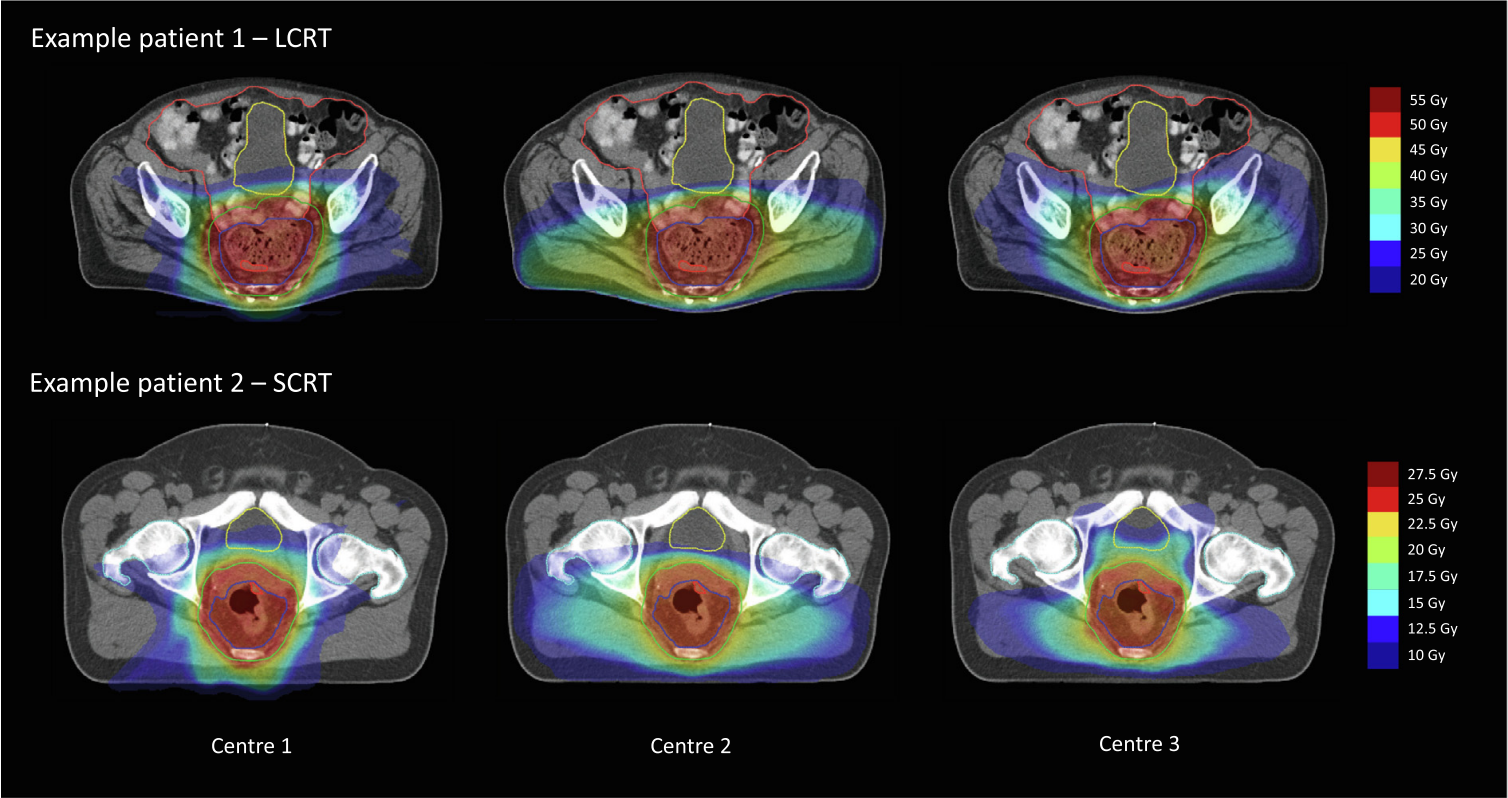
Between-centre variation in dose planning, for patients with limited and larger variation in organ at risk (OAR) dose metrics. Short-course radiotherapy (SCRT, 25 Gy/5 fractions) and long-course radiotherapy (LCRT, 50 Gy/25 fractions) chosen for illustration, not to indicate that either of the treatment schedules demonstrated more planning heterogeneity. Treatment planning details for each centre are described in the main text. Red outline: Gross tumour volume, GTV. Dark blue outline: Clinical target volume, CTV. Green outline: Planning target volume, PTV. Orange outline: Bowel cavity. Yellow outline: Bladder. Light blue outline: Femoral heads. (For interpretation of the references to colour in this figure legend, the reader is referred to the web version of this article.)

**Fig. 2:**
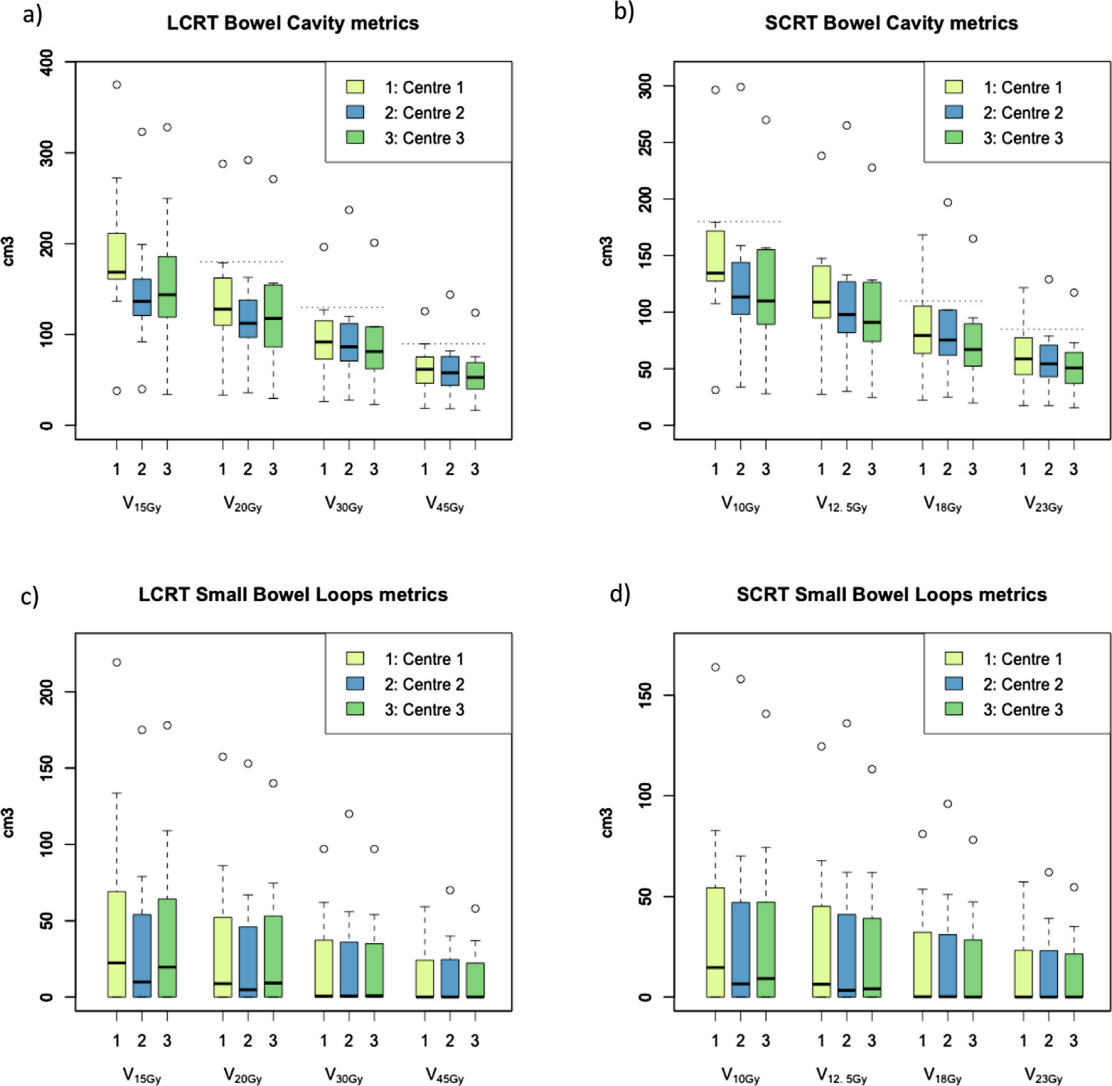
Boxplots of dose metrics for bowel cavity (a and b) and small bowel loops (c and d) for long- and short-course mesorectal-only radiotherapy. Dotted lines indicate optimisation objectives as suggested in Table 2. Circles represent outliers outside 1.5 times the interquartile range (IQR).

**Fig. 3:**
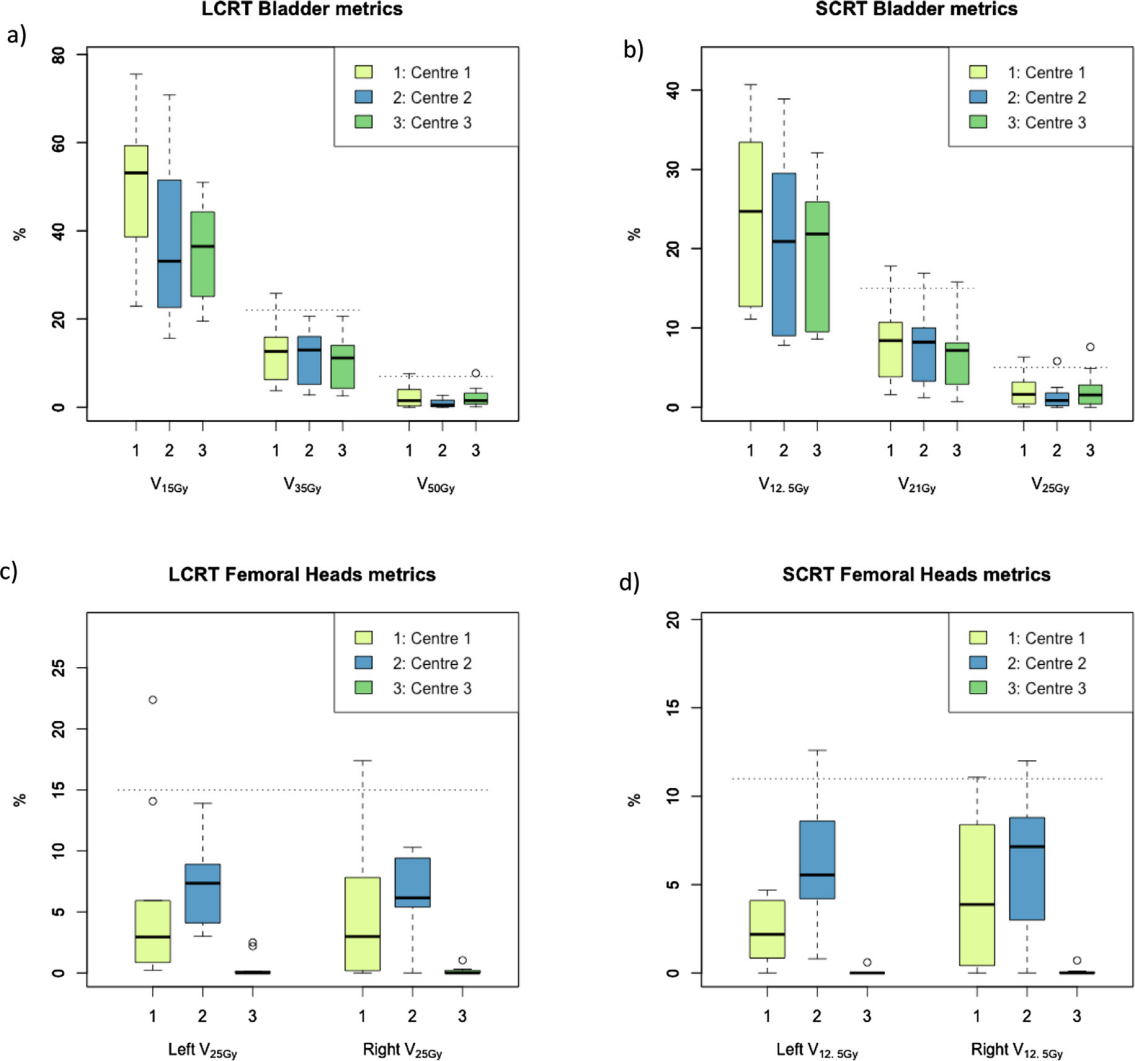
Boxplots of dose metrics for bladder (a and b) and femoral heads (c and d) for long- and short-course mesorectal-only radiotherapy. Dotted lines indicate optimisation objectives as suggested in Table 2. Note that centre 2 did not spare the dose to the femoral heads as much as possible, the suggested objective for femoral heads was only added if necessary.

**Fig. 4:**
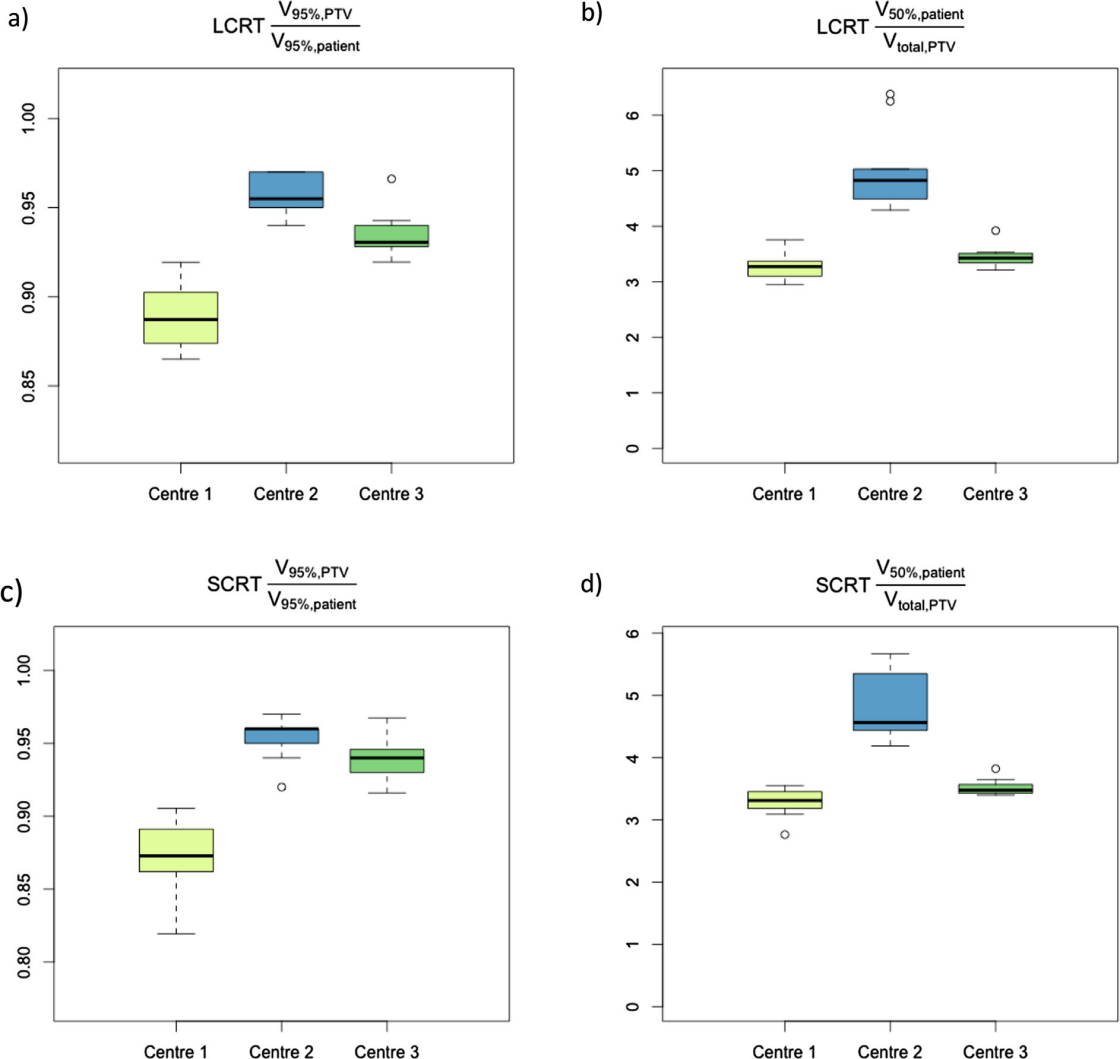
Boxplots of conformity indices (CI1 and CI2, see main text) for high dose to the PTV (a and b) and spill-over of median dose levels into surrounding normal tissue (c and d) for long- and short-course mesorectal-only radiotherapy. PTV: Planning target volume.

Dose metrics were generally consistent across centres, with the main variation seen for low dose bowel and bladder metrics, femoral heads doses, and CI2 (relative proportion between volume receiving 50% of prescription dose and total volume of PTV). Fig. 1 illustrates some of the planning variation seen for two example patients, mainly related to lateral dose spill to spare anterior structures. See Fig. 2, Fig. 3, Fig. 4 for visual illustration of the main results.

Based on these results, optimisation objectives were identified and validated across centres; summarised in Table 2. As described, these objectives are chosen to be achievable for at least 80% (bowel) or 90% (bladder, femoral heads) in an individual centre. On multi-centre validation, all objectives were achievable for 90% of plans overall. Femoral head objectives are valid for individual (left/right) femoral heads as well as for the combined volume.

**Table 2:** Suggested optimisation objectives for long-course (LCRT) and short-course (SCRT) mesorectal-only radiotherapy. Femoral head objectives are valid for individual (left/right) femoral heads as well as for the combined volume.

	Optimisation objectives	
Organ at risk	*LCRT (25 × 2Gy)*	*SCRT (5 × 5Gy)*
Bowel cavity	*V*_20Gy_ < 180 cm^3^	*V*_10Gy_ < 180 cm^3^
	*V*_30Gy_ < 130 cm^3^	*V*_18Gy_ < 110 cm^3^
	*V*_45Gy_ < 90 cm^3^	*V*_23Gy_ < 85 cm^3^

Bladder	*V*_35Gy_ < 22%	*V*_21Gy_ < 15%
	*V*_50Gy_ < 7%	*V*_25Gy_ < 5%

Femoral heads	*V*_25Gy_ < 15%	*V*_12.5Gy_ < 11%

Small bowel loop dose metrics demonstrated large variation between patients, compared to bowel cavity; many patients had zero volume of small bowel loops irradiated across most dose levels. Detailed results for all evaluated dose metrics are presented in Tables C1 and C2 (Appendix C, supplementary materials, online only).

## Discussion

Our multicentre planning study has identified robust dose planning objectives appropriate for mesorectal-only radiotherapy. These objectives have been chosen to be achievable for the majority of patients planned using modern, intensity modulated treatment techniques. Generally, there is limited clinical evidence available to guide the choice of dose levels for plan optimisation in rectal cancer. Given the lack of published data, we used a combination of literature review, expert opinion, and best practice in academic centres to arrive at pragmatic dose planning objectives. These now form the basis of the radiotherapy planning recommendations in the STAR-TREC trial.

Short course radiotherapy or chemoradiotherapy may be considered for patients with early rectal cancer eligible for an organ preserving approach. In this setting, mesorectal radiotherapy is attractive, since it is targeted to the primary tumour and the surrounding tissue at risk for involved lymph nodes, minimising toxicity and optimising chances for acceptable functional outcome and quality of life.

To fully employ the benefits of this reduced target volume, high-quality, conformal radiotherapy is warranted. The literature available to inform plan optimisation guidelines is sparse, however, and much of it is from 2D and 3D-CRT treatment era. Data from 3D-CRT should be used with caution in the IMRT setting, as close correlation between dose metrics in the 15–50 Gy range for 3D-CRT treatment plans can make it difficult to elucidate any high- and medium-range dose effect. OAR outlining conventions, chemotherapy schedules, and supportive care may also have changed, making it unlikely that specific dose constraints based on non-contemporary patient series may be directly applicable to modern rectal cancer treatment. Even though absolute dose constraints for prevention of treatment-related toxicity might not be known, however, objectives can be established that at least ensure that plans are optimised and conformal compared to a representative patient cohort. This paper provides such approach, based on a multicentre treatment planning study. The methodology presented here could be relevant for other multicentre trials, and could thus be of general interest outside the rectal cancer organ preservation space.

The suggested objectives should ensure a reasonable plan quality for most patients, although individual patients can likely be optimised further [32]. We deliberately included a range of patient cases, to ensure that results are robust for factors known to impact OAR doses in rectal cancer radiotherapy, such as tumour height, patient positioning [33] and gender [34]. Thus objectives should be achievable for most patient groups and across treatment planning systems; although centres might consider tighter objectives if consistently achievable for their local patients. Additionally, the optimisation objectives still leave room for plan variation, as demonstrated in our data. No optimisation objectives are suggested for low dose levels (<15 Gy for LCRT, <10 Gy for SCRT), and there might be resulting variation in clinical planning practice, especially depending on local beam setups (choice of beam angles for IMRT, partial versus full arcs for arc therapy). The variations in CI1 and CI2 across centres illustrate this point: Differences might be explained by disparate prioritisation of e.g. anterior dose spill-over, high dose conformality, hotspots in OARs, etc, in the plan optimisation process. Individual clinical teams will likely want to assess additional plan metrics as part of their plan approval and prescription process. At this point, we are unable to identify evidence for specific assessment criteria, however.

Other limitations of the current study include the small number of treatment planners and systems used; ideally all major commercial treatment planning systems should have been included, with multiple planners and/or institutions per system. The STAR-TREC trial will allow retrospective plan review across centres and systems. Comparison of results from arc therapy (used by all centres in the study) with IMRT would have been interesting, but likely of limited practical use, as arc therapy is the dominating technique for intensity modulated delivery in current clinical practice. This situation could change in the future, as commercial MR-guided radiotherapy (MRgRT) systems use step-and-shot IMRT for treatment delivery. MRgRT IMRT appears to provide a slightly worse plan quality compared to conventional linac-based VMAT for rectal cancer [35], and the current study results may thus not be applicable in this setting. Conversely, MRgRT could potentially allow for treatment margin reduction: The current study used large, but appropriate, CTV-to-PTV margins [36], which can very likely be reduced with daily (MRI) guidance and adaptive strategies. If that becomes the case, it might be necessary to re-evaluate the suggested planning objectives to ensure optimal plan quality across future patient populations.

The current study focused on “classic” pelvic OARs for rectal cancer radiotherapy; bowel, bladder and femoral heads. In the organ preservation setting, other normal tissue may emerge as more relevant for long term functional outcomes and quality of life. These could include the anal sphincter and pelvic floor muscles [37], [38], pelvic bones [39], and vagina [40] or penile bulb. There is very little published data optimising rectal cancer radiotherapy in the organ preservation setting, and basic questions around relevant patient-experienced toxicity endpoints and OARs still need to be addressed. High quality clinical data, preferably from prospective trials, are needed to guide further development.

## Conclusion

Introduction of radiotherapy regimen and volumes in novel settings can prove challenging, as limited clinical data are available to guide plan optimisation. We utilized a multicentre planning study approach to develop robust planning objectives for mesorectal radiotherapy for early rectal cancer. The suggested objectives should support the safe implementation of the novel mesorectal treatment volume in prospective trials, e.g. as currently used in the STAR-TREC trial.

## Funding

The STAR-TREC trial is funded in the UK by Cancer Research UK (C41557/A19393), in the Netherlands by the Dutch Cancer Society (KWF KUN 2014-7448), and in Denmark by the Danish Cancer Society (R100-A6747). AA is supported by Yorkshire Cancer Research Academic Fellowship funding (grant L389AA). MT was supported by Academy of Medical Sciences Starter Grant for Clinical Lecturers. None of the funders have had any involvement in the study design; in the collection, analysis and interpretation of data; in the writing of the report; or in the decision to submit the article for publication.

## Declaration of Competing Interest

The authors declare that they have no known competing financial interests or personal relationships that could have appeared to influence the work reported in this paper.
