# Supplementary material for: Robust dose planning objectives for mesorectal radiotherapy of early stage rectal cancer – A multicentre dose planning study
Source: Tech Innov Patient Support Radiat Oncol. 2019 Oct 15;11:14–21. doi: 10.1016/j.tipsro.2019.09.001 (PMC7033757; doi:10.1016/j.tipsro.2019.09.001)
Supplement: Supplementary Data 1 [file mmc1.pdf]

# Supplementary materials

## Robust dose planning objectives for mesorectal radiotherapy of early stage rectal cancer – a multicentre dose planning study

## Appendix A: Selected studies of dose-volume effect for bowel toxicity

| **Reference** | **Endpoint** | **Type** | **Patient #** | **Patient population** | **Treatment technique** | **OAR definition** | **Main findings** |
|---|---|---|---|---|---|---|---|
| Baglan et al (1) | Acute bowel toxicity | Retrospective | 40 | Rectal cancer, LCRT | 3D-CRT | Bowel loops | Correlation with volume at each dose level at 5-40 Gy. Suggestion of "threshold effect" at ~15-25 Gy. Suggested volume-response for $V_{15Gy}$. |
| Tho et al (2) | Acute bowel toxicity | Retrospective | 41 | Rectal cancer, LCRT | 3D-CRT | "Small bowel" (loops?) | Correlation with volume at each dose level at 5-40 Gy. Strongest correlations for $V_{5Gy}$ -$V_{30Gy}$ |
| Gunnlaugsson et al (3) | Acute bowel toxicity | Retrospective | 28 | Rectal cancer, LCRT + oxaliplatin | 3D-CRT | Small bowel loops, large bowel, whole abdomen | Only correlation for volume of small bowel irradiated. Correlation with all dose levels 5-50 Gy, strongest for $V_{15Gy}$. |
| Kavanagh et al (4) | Acute & late bowel toxicity | **Review** (QUANTEC) | NA | Various pelvic cancers | Primarily 3D-CRT | NA | Six studies of small bowel complication identified for review of acute toxicity; one study for late toxicity. Conclusion: Outlining of bowel loops - minimise $V_{15Gy}$. Outlining peritoneal space - minimise $V_{45Gy}$. This will also likely reduce late toxicity risk. |
| Robertson et al (5) | Acute bowel toxicity | Retrospective | 152 | Rectal cancer, LCRT pre- & post-op | 3D-CRT | Small bowel loops | Absolute $V_{15Gy}$, $V_{20Gy}$ and $V_{25Gy}$ correlated with acute toxicity; similar findings for pre- and post-operative patients. |
| Chen et al (6) | Patient-reported acute GI symptoms | Prospective PRO collection, explorative analysis | 66 | Rectal cancer, LCRT | 3D-CRT | Small bowel loops | Absolute $V_{15Gy}$ correlated with overall patient-reported GI symptoms. |
| Banerjee et al (7) | Acute bowel toxicity | Retrospective | 67 | Rectal cancer, LCRT | 3D-CRT | Small bowel loops, peritoneal space | Absolute $V_{15Gy}$ and $V_{25Gy}$ correlated with acute toxicity for both small bowel loops and peritoneal space. |

| | | | | | | | |
|---|---|---|---|---|---|---|---|
| Yang et al (8) | Acute bowel toxicity | Retrospective | 177 | Rectal cancer, LCRT | IMRT & 3D-CRT | Bowel loops | Relative $V_{45Gy}$ best predictor for acute toxicity in multivariate modelling. |
| Devisetty et al (9) | Acute bowel toxicity | Retrospective | 48 | Anal cancer, CRT | IMRT | Outer contour of bowel | Absolute $V_{30Gy}$ correlated with clinically significant acute toxicity. |
| DeFoe et al (10) | Acute bowel toxicity | Retrospective | 58 | Anal cancer, CRT | IMRT | Intestinal cavity | Absolute $V_{30Gy}$ and $V_{40Gy}$ correlated with acute toxicity. |
| Han et al (11) | Acute bowel toxicity | Prospective | 42 | Anal cancer, CRT | IMRT | Small and large bowel loops | Relative $V_{30Gy}$ for small bowel showed moderate correlation with acute toxicity; no correlation for large bowel. |
| Julie et al (12) | Acute bowel toxicity | Retrospective | 108 | Anal cancer, CRT | IMRT | Bowel loops | Model combining absolute bowel $V_{45Gy}$ and mean dose to hottest 5% of bowel best predictor for acute toxicity. |
| Olsen et al (13) | Acute & late bowel toxicity | Prospective | 52 | Anal cancer, CRT | IMRT | Small bowel loops, intestinal cavity | Absolute $V_{25Gy}$-$V_{35Gy}$ for small bowel correlated with acute toxicity (and absolute $V_{5Gy}$-$V_{25Gy}$ for intestinal cavity with high grade acute toxicity). Absolute $V_{60Gy}$ for intestinal cavity correlated with late toxicity. |
| Ng et al (14) | Acute bowel toxicity | Retrospective | 55 | Anal cancer, CRT | IMRT | Small and large bowel loops, intestinal cavity | Absolute volume of intestinal cavity correlated with acute toxicity for all dose levels 5-50 Gy; strongest correlation for $V_{10Gy}$-$V_{35Gy}$. |
| Sanguineti et al (15) | Acute bowel toxicity | Retrospective | 149 | Prostate cancer | 3D-CRT & IMRT | Intestinal cavity | Absolute $V_{15Gy}$ and $V_{30Gy}$ cavity correlated with acute toxicity. |
| Perna et al (16) | Acute bowel toxicity | Retrospective | 96 | Prostate cancer, post-op | IMRT | Intestinal cavity outside PTV, bowel loops | Relative $V_{50Gy}$ for bowel loops correlated with acute toxicity. |
| Sina et al (17) | Patient-reported acute GI symptoms | Prospective | 206 | Prostate cancer, some post-op | IMRT | Small and large bowel loops | Best multivariate model for change in loose stools from baseline to RT mid-point included absolute $V_{42Gy}$ for small bowel. |
| Chi et al (18) | Acute bowel toxicity | Retrospective | 32 | Endometrial cancer, post-op | IMRT | Small bowel loops, limited bowel space, intestinal cavity | Relative $V_{45Gy}$ for intestinal cavity correlated with acute toxicity, no correlation for alternative outlining. |
| Isohashi et al (19) | Acute & late bowel toxicity | Retrospective | 62 | Cervical cancer, post-op | 3D-CRT & IMRT | Small bowel loops, bowel bag | Higher values of absolute $V_{40Gy}$ and $V_{45Gy}$ for both small bowel and bowel bag for patients with acute toxicity, |

| | | | | | | | |
|---|---|---|---|---|---|---|---|
| | | | | | | | higher values of absolute $V_{15Gy}$-$V_{45Gy}$ for patients with chronic toxicity. |
| Chopra et al (20) | Acute & late bowel toxicity | Retrospective | 103 | Cervical cancer, post-op | 3D-CRT & IMRT | Small and large bowel loops, intestinal cavity | Absolute $V_{30Gy}$ for intestinal cavity correlated with acute toxicity. Absolute $V_{30Gy}$-$V_{40Gy}$ for small and large bowel and intestinal cavity correlated with late toxicity in univariate, but not multivariate, analysis. |

## Pubmed search string

> *((((chemoradiation) OR radiation therapy) OR radiochemotherapy)) AND ((((Toxicity intestines) OR (((Gastrointestinal toxicity) OR Gastrointestinal side effects)))) OR Side effects intestines) AND ((dose-response) OR dose-volume)*

Abstracts were screened by hand, and relevant papers selected for full text review, with additional studies identified from reference lists.

## References


1. Baglan KL, Frazier RC, Yan D, *et al.* The dose-volume relationship of acute small bowel toxicity from concurrent 5-FU-based chemotherapy and radiation therapy for rectal cancer. *Int. J. Radiat. Oncol. Biol. Phys.* 2002;52:176–83.
2. Tho LM, Glegg M, Paterson J, *et al.* Acute small bowel toxicity and preoperative chemoradiotherapy for rectal cancer: investigating dose-volume relationships and role for inverse planning. *Int. J. Radiat. Oncol. Biol. Phys.* 2006;66:505–13.
3. Gunnlaugsson A, Kjellén E, Nilsson P, *et al.* Dose-volume relationships between enteritis and irradiated bowel volumes during 5-fluorouracil and oxaliplatin based chemoradiotherapy in locally advanced rectal cancer. *Acta Oncol.* 2007;46:937–44.
4. Kavanagh BD, Pan CC, Dawson LA, *et al.* Radiation dose-volume effects in the stomach and small bowel. *Int. J. Radiat. Oncol. Biol. Phys.* 2010;76:S101-7.
5. Robertson JM, Söhn M, Yan D. Predicting grade 3 acute diarrhea during radiation therapy for rectal cancer using a cutoff-dose logistic regression normal tissue complication probability model. *Int. J. Radiat. Oncol. Biol. Phys.* 2010;77:66–72.
6. Chen RC, Mamon HJ, Ancukiewicz M, et al. Dose-volume effects on patient-reported acute gastrointestinal symptoms during chemoradiation therapy for rectal cancer. Int J Radiat Oncol Biol Phys 2012;83:e513-7
7. Banerjee R, Chakraborty S, Nygren I, *et al.* Small bowel dose parameters predicting grade ≥ 3 acute toxicity in rectal cancer patients treated with neoadjuvant chemoradiation: an independent validation study comparing peritoneal space versus small bowel loop contouring techniques. *Int. J. Radiat. Oncol. Biol. Phys.* 2013;85:1225–31.
8. Yang TJ, Oh JH, Son CH, *et al.* Predictors of acute gastrointestinal toxicity during pelvic chemoradiotherapy in patients with rectal cancer. *Gastrointest. Cancer Res.* 2013;6:129–36.

9. Devisetty K, Mell LK, Salama JK, *et al.* A multi-institutional acute gastrointestinal toxicity analysis of anal cancer patients treated with concurrent intensity-modulated radiation therapy (IMRT) and chemotherapy. *Radiother. Oncol.* 2009;93:298–301.
10. DeFoe SG, Kabolizadeh P, Heron DE, *et al.* Dosimetric parameters predictive of acute gastrointestinal toxicity in patients with anal carcinoma treated with concurrent chemotherapy and intensity-modulated radiation therapy. *Oncology*. 2013;85:1–7.
11. Han K, Cummings BJ, Lindsay P, *et al.* Prospective evaluation of acute toxicity and quality of life after IMRT and concurrent chemotherapy for anal canal and perianal cancer. *Int. J. Radiat. Oncol. Biol. Phys.* 2014;90:587–594.
12. Julie DAR, Oh JH, Apte AP, *et al.* Predictors of acute toxicities during definitive chemoradiation using intensity-modulated radiotherapy for anal squamous cell carcinoma. *Acta Oncol.* 2016;55:208–16.
13. Olsen JR, Moughan J, Myerson R, *et al.* Predictors of Radiation Therapy–Related Gastrointestinal Toxicity From Anal Cancer Dose-Painted Intensity Modulated Radiation Therapy: Secondary Analysis of NRG Oncology RTOG 0529. *Int. J. Radiat. Oncol. Biol. Phys.* 2017;98:400–408.
14. Ng M, Ho H, Skelton J, et al. Intensity-modulated Radiotherapy for Anal Cancer: Dose-Volume Relationship of Acute Gastrointestinal Toxicity and Disease Outcomes. Clin Oncol 2018;30(10):634-641
15. Sanguineti G, Endres EJ, Sormani MP, *et al.* Dosimetric predictors of diarrhea during radiotherapy for prostate cancer. *Strahlenther. Onkol.* 2009;185:390–6.
16. Perna L, Alongi F, Fiorino C, *et al.* Predictors of acute bowel toxicity in patients treated with IMRT whole pelvis irradiation after prostatectomy. *Radiother. Oncol.* 2010;97:71–5.
17. Sini C, Noris Chiorda B, Gabriele P, *et al.* Patient-reported intestinal toxicity from whole pelvis intensity-modulated radiotherapy: First quantification of bowel dose–volume effects. *Radiother. Oncol.* 2017;124:296–301.
18. Chi A, Nguyen NP, Xu J, *et al.* Correlation of three different approaches of small bowel delineation and acute lower gastrointestinal toxicity in adjuvant pelvic intensity-modulated radiation therapy for endometrial cancer. *Technol. Cancer Res. Treat.* 2012;11:353–359.
19. Isohashi F, Mabuchi S, Yoshioka Y, *et al.* Intensity-modulated radiation therapy versus three-dimensional conformal radiation therapy with concurrent nedaplatin-based chemotherapy after radical hysterectomy for uterine cervical cancer: comparison of outcomes, complications, and dose-volume histogr. *Radiat. Oncol.* 2015;10:180.
20. Chopra S, Krishnatry R, Dora T, *et al.* Predictors of late bowel toxicity using three different methods of contouring in patients undergoing postoperative radiation for cervical cancer. *Br. J. Radiol.* 2015;88.

## Appendix B: Calculation of SCRT optimisation dose levels

All short course radiotherapy (SCRT) optimisation dose levels were based on dose levels from long course radiotherapy (LCRT), recalculated to 5-fraction treatment schedules, using:

$$D_{SCRT} = \frac{n_{SCRT}}{2}\left(\sqrt{\left({}^{\alpha}/_{\beta}\right)^2 + 4\frac{D_{LCRT}}{n_{SCRT}}\left(\frac{D_{LCRT}}{n_{LCRT}} + {}^{\alpha}/_{\beta}\right)} - {}^{\alpha}/_{\beta}\right)$$

where $D_{SCRT}$ / $n_{SCRT}$ and $D_{SCRT}$ / $n_{SCRT}$ are total dose and number of fractions for the SCRT and LCRT treatment schedules, respectively. This equation follows directly from the linear-quadratic equation for effective dose.

Dose levels were recalculated using $\alpha/\beta$ = 10 Gy (generic value for acute complications) and $\alpha/\beta$ = 3 Gy (generic value for late complications), and with $n_{SCRT}$ set to 25-30 fractions (to represent the range of LCRT schedules used in clinical practice).

Results can be found in Table B1.

| | | **α/β = 10 Gy** | **α/β = 3 Gy** |
|---|---|---|---|
| **15 Gy** | | | |
| | Originally in 25 fractions | 12.7 Gy | 10.6 Gy |
| | Originally in 30 fractions | 12.6 Gy | 10.4 Gy |
| **30 Gy** | | | |
| | Originally in 25 fractions | 23.0 Gy | 18.7 Gy |
| | Originally in 30 fractions | 22.7 Gy | 18.1 Gy |
| **35 Gy** | | | |
| | Originally in 25 fractions | 26.2 Gy | 21.2 Gy |
| | Originally in 30 fractions | 25.8 Gy | 20.5 Gy |

**Table B1:** Recalculation of dose levels from long course (25-30 fractions) to short course (5 fractions) schedules.

## Appendix C: Detailed dose planning results

| Planning results long-course radiotherapy | | | |
|---|---|---|---|
| | **Centre 1** | **Centre 2** | **Centre 3** |
| CTV | | | |
| $V_{47.5Gy}$ [%] | 100.0 (100.0 -100.0) | 100.0 (100.0 - 100.0) | 100.0 (100.0 - 100.0) |
| PTV | | | |
| $V_{47.5Gy}$ [%] | 99.4 (99.1 - 99.4) | 99.1 (99.1 - 99.2) | 99.8 (99.7 - 99.9) |
| $V_{45Gy}$ [%] | 100.0 (100.0 - 100.0) | 100.0 (100.0 - 100.0) | 100.0 (100.0 - 100.0) |
| $V_{52.5Gy}$ [%] | 0.1 (0.1 - 0.3) | 0.0 (0.0 – 0.0) | 0.0 (0.0 – 0.0) |
| $D_{50\%}$ [Gy] | 50.5 (50.4 - 50.5) | 49.8 (49.7 - 49.9) | 50.0 (50.0 - 50.0) |
| Bowel cavity | | | |
| $V_{15Gy}$ [$cm^3$] | 169 (162 – 201) | 137 (123 – 158) | 144 (121 – 185) |
| $V_{20Gy}$ [$cm^3$] | 128 (111 – 156) | 113 (98 – 136) | 118 (89 – 153) |
| $V_{30Gy}$ [$cm^3$] | 92 (74 – 111) | 87 (72 – 109) | 81 (63 – 108) |
| $V_{45Gy}$ [$cm^3$] | 62 (47 – 73) | 58 (45 – 75) | 53 (41 – 68) |
| Small bowel loops | | | |
| $V_{15Gy}$ [$cm^3$] | 22 (0 – 68) | 10 (0 – 45) | 20 (0 – 57) |
| $V_{20Gy}$ [$cm^3$] | 9 (0 – 47) | 5 (0 – 37) | 9 (0 – 45) |
| $V_{30Gy}$ [$cm^3$] | 1 (0 – 30) | 1 (0 – 28) | 1 (0 – 27) |
| $V_{45Gy}$ [$cm^3$] | 0 (0 – 18) | 0 (0 – 19) | 0 (0 – 17) |
| Bladder | | | |
| $V_{15Gy}$ [%] | 53.1 (39.4 - 58.4) | 33.1 (22.7 - 50.6) | 36.5 (25.3 - 43.1) |
| $V_{35Gy}$ [%] | 12.6 (6.4 - 15.3) | 13.0 (5.5 - 15.8) | 11.2 (4.5 - 13.6) |
| $V_{50Gy}$ [%] | 1.5 (0.5 - 3.5) | 0.5 (0.1 - 1.4) | 1.5 (0.8 - 3.0) |
| Right femoral head | | | |
| $V_{25Gy}$ [%] | 3.0 (0.3 - 7.4) | 6.2 (5.4 - 9.1) | 0.0 (0.0 - 0.2) |
| $V_{50Gy}$ [%] | 0.0 (0.0 - 0.0) | 0.0 (0.0 - 0.0) | 0.0 (0.0 - 0.0) |
| Left femoral head | | | |
| $V_{25Gy}$ [%] | 2.9 (0.9 - 5.7) | 7.4 (4.3 - 8.6) | 0.0 (0.0 - 0.1) |
| $V_{50Gy}$ [%] | 0.0 (0.0 - 0.0) | 0.0 (0.0 - 0.0) | 0.0 (0.0 – 0.0) |
| CI1 | 0.89 (0.87 - 0.90) | 0.96 (0.95 - 0.97) | 0.93 (0.93 - 0.94) |
| CI2 | 3.27 (3.11 - 3.37) | 4.83 (4.53 - 5.02) | 3.43 (3.34 - 3.50) |

**Table C1:** Planning results for long-course radiotherapy (LCRT) divided by centre. All numbers are medians with interquartile ranges (IQR) in brackets. CTV: Clinical target volume. PTV: Planning target volume. CI: Conformity index.

| Planning results short-course radiotherapy | | | |
|---|---|---|---|
| | **Centre 1** | **Centre 2** | **Centre 3** |
| CTV | | | |
| $V_{23.75Gy}$ [%] | 100.0 (100.0 - 100.0) | 100.0 (100.0 - 100.0) | 100.0 (100.0 - 100.0) |
| PTV | | | |
| $V_{23.75Gy}$ [%] | 99.4 (99.3 - 99.5) | 99.1 (99.0 - 99.1) | 99.8 (99.5 - 99.8) |
| $V_{22.5Gy}$ [%] | 100.0 (99.9 - 100.0) | 100.0 (100.0 - 100.0) | 100.0 (100.0 - 100.0) |
| $V_{26.25Gy}$ [%] | 0.2 (0.1 - 0.3) | 0.0 (0.0 - 0.0) | 0.7 (0.4 - 0.8) |
| $D_{50}$ [Gy] | 25.3 (25.2 - 25.3) | 24.9 (24.9 - 24.9) | 25.0 (25.0 - 25.0) |
| Bowel cavity | | | |
| $V_{10Gy}$ [$cm^3$] | 135 (128 – 168) | 114 (102 – 142) | 110 (92 – 155) |
| $V_{12.5Gy}$ [$cm^3$] | 109 (96 – 138) | 98 (84 – 125) | 91 (75 – 126) |
| $V_{18Gy}$ [$cm^3$] | 79 (65 – 101) | 76 (63 – 99) | 67 (53 – 88) |
| $V_{23Gy}$ [$cm^3$] | 59 (46 – 74) | 55 (43 - 70) | 51 (38 – 64) |
| Small bowel loops | | | |
| $V_{10Gy}$ [$cm^3$] | 15 (0 – 51) | 7 (0 – 39) | 9 (0 – 40) |
| $V_{12.5Gy}$ [$cm^3$] | 6 (0 – 38) | 3 (0 – 32) | 4 (0 – 32) |
| $V_{18Gy}$ [$cm^3$] | 0 (0 – 25) | 0 (0 – 24) | 0 (0 – 22) |
| $V_{23Gy}$ [$cm^3$] | 0 (0 – 17) | 0 (0 – 17) | 0 (0 – 16) |
| Bladder | | | |
| $V_{12.5Gy}$ [%] | 24.7 (14.3 - 33.1) | 20.9 (9.4 - 28.9) | 21.9 (9.7 - 25.5) |
| $V_{21Gy}$ [%] | 8.4 (3.9 - 10.6) | 8.2 (3.6 - 9.8) | 7.2 (3.1 - 8.1) |
| $V_{25Gy}$ [%] | 1.6 (0.6 - 3.0) | 0.9 (0.3 - 1.8) | 1.6 (0.6 - 2.8) |
| Right femoral head | | | |
| $V_{12.5Gy}$ [%] | 3.9 (0.7 - 7.9) | 7.2 (3.1 - 8.8) | 0.0 (0.0 - 0.0) |
| $V_{25Gy}$ [%] | 0.0 (0.0 - 0.0) | 0.0 (0.0 - 0.0) | 0.0 (0.0 - 0.0) |
| Left femoral head | | | |
| $V_{12.5Gy}$ [%] | 2.2 (1.1 - 3.9) | 5.6 (4.4 - 8.2) | 0.0 (0.0 – 0.0) |
| $V_{25Gy}$ [%] | 0.0 (0.0 - 0.0) | 0.0 (0.0 - 0.0) | 0.0 (0.0 - 0.0) |
| CI1 | 0.87 (0.86 - 0.89) | 0.95 (0.95 - 0.96) | 0.94 (0.93 - 0.95) |
| CI2 | 3.31 (3.20 - 3.45) | 4.57 (4.45 - 5.17) | 3.48 (3.44 - 3.56) |

**Table 2:** Planning results for short-course radiotherapy (SCRT) divided by centre. All numbers are medians with interquartile ranges (IQR) in brackets. CTV: Clinical target volume. PTV: Planning target volume. CI: Conformity index.
